# Child Slaves of Louisiana

**Published:** 1912-05

**Authors:** 


### Child Slaves of Louisiana.

Dr. Mayer Newhauser, the medical inspector attached to the Louisiana State Board of Health, has described the canning factory at Violet, La., as "hell itself." The conditions there, where little boys and girls at ages between 6 and 8 are working out their little bodies for a corporation, their poor little hands bearing mute testimony in the form of cuts and hypertrophies to the hardships to which they are exposed must pass belief. According to the medical inspector's report the insanitary conditions are, strictly speaking, unprintable. "The five-room rickety wooden structure which serves as toilet accommodations have no seats within, but a plain running-board is used by men, women and children, and with its coincident accumulation of feces, which appear to have been there since the toilet was erected, and the horrible stench arising therefrom, causes us to shrink involuntarily from such sordid surroundings, the like of which is hardly believable in this age of civilization. From the toilet back to the shucking of oysters and placing them in tin cans, without washing hands, is a most common occurrence. All the operatives, including the little children, are exceedingly dirty; yet how can they be otherwise when there is no provision for washing?" The inspector calls upon the club women of the State to give up for a moment their political debates, and to listen to the cries of the child slaves of Louisiana? One hears of such things almost with incredulity, for society can not so far forget its humanitarian instincts in this enlightened age to permit such horrible condition to exist; yet the facts as stated have been verified by the Louisiana State Board of Health. The conditions are a sad commentary on our boasted age, which aims to civilize the heathen in foreign countries, but forgets the heathen in high places at home.—*Lancet-Clinic.*

["The inspector calls upon the club women of the State," etc. Why he should do so,—why give this gratuitous slap at the women, is surprising. *They* have no authority under law,—no power, no voice in making sanitary or other laws. On the contrary, it is by law made specially the duty of the Board of Health to do that especial work. To find out the need of such action, he, Mr. Inspector, is paid and sent out. Dr. Dowling, President of the Board, has toured the country in a special train of cars in search of just such sanitary evils, and has had more advertising than any man alive, and yet here, right under his nose, is a most pitiful, most flagrant violation of all sanitation, and this Mr. Inspector holds his nose and calls on the club women to "give up their political debates and listen to the cries of these children." They will continue to cry until the Board does its duty.—D.]

